# The use of spent coffee grounds and spent green tea leaves for the removal of cationic dyes from aqueous solutions

**DOI:** 10.1038/s41598-021-89095-6

**Published:** 2021-05-05

**Authors:** Tomasz Jóźwiak, Urszula Filipkowska, Joanna Struk-Sokołowska, Kamil Bryszewski, Karol Trzciński, Joanna Kuźma, Monika Ślimkowska

**Affiliations:** 1 Department of Environmental Engineering, University of Warmia and Mazury in Olsztyn, Warszawska St. 117a, 10-957 Olsztyn, Poland; 2 Department of Environmental Engineering Technology, Bialystok University of Technology, Wiejska St. 45E, 15-351 Bialystok, Poland

**Keywords:** Environmental chemistry, Chemical engineering

## Abstract

This study aimed to examine sorption effectiveness of cationic dyes: Basic Red 46 (BR46) and Basic Violet 10 (BV10) onto spent coffee ground (CG) and spent green tea leaves (GTL). The scope of the study included, i.a.: sorbent FTIR spectra analysis, determination of pH effect on dye sorption effectiveness, analysis of dye sorption kinetics, and determination of maximal sorption capacity of the sorbents. The effectiveness of BR46 sorption on the sorbents tested was the highest at pH 6 and that of BV10 at pH 3. Both sorbents caused changes in solution pH during the sorption process, due to the system tending to reach the pH value approximating the pH_ZPC_ (pH_PZC_ = 7.55 for CG and pH_PZC_ = 7.05 for GTL). The time needed to reach BR46 and BV10 sorption equilibrium onto CG and GTL ranged from 180 to 240 min. The intramolecular diffusion model demonstrated that the sorption of cationic dyes onto CG and GTL proceeded in three phases differing in the intensity and duration. The maximal sorption capacity of CG reached 179.4 mg/g for BR46 and 59.3 mg/g for BV10. The sorption capacity of GTL was lower and reached 58.0 mg/g for BR46 and 26.7 mg/g for BV10.

## Introduction

Post-production wastewater from the textile, tanning, or paper industries can contain significant amounts of dyes. Part of these dyes can pervade the natural environment because of the still imperfect technologies for wastewater decolorization. Dyes present in natural waters block the access of sun-rays to aquatic plants, thus inhibiting the photosynthesis process. In this respect, the most adverse to the natural environment seem to be cationic dyes, as even in very minor quantities (< 1 mg/mL), they effectively colorize water, significantly decreasing its transparency. The reduction of the primary production and oxygen synthesis by autotrophs can lead to the aquatic ecosystem’s breakdown. Therefore, possibly the most effective methods for dye removal from wastewater should be employed to prevent the risk of environmental degradation. Currently produced synthetic dyes are sparingly biodegradable; hence industrial wastewater containing these dyes is rarely treated using biological methods. The decolorization of post-production waters is usually conducted with various physicochemcial methods, like e.g., oxidation, coagulation, membrane processes, or sorption. It is commonly believed today that dye sorption offers one of the simplest and safest methods for wastewater decolorization.

The sorption process consists in binding one substance (sorbate) by another substance (sorbent). Its costs depend primarily on sorbent price, while its effectiveness is largely determined by sorbent type. The most common commercial sorbents include materials based on activated carbons, which exhibit a high sorption effectiveness toward most of the dyes available on the market. The good sorption properties of these materials are due to, i.a., very large specific surface area, approximating 1000 m^2^/g. Activated carbons are usually sourced from solid fossil fuels or lignocellulosic plant biomass. Because of their stepwise and energy-consuming production (dehydration, carbonization, activation), they are usually very costly, which is their greatest drawback. This prompts the search for their cheaper substitutes.

Today, the raw materials for the production of unconventional sorbents are most often searched for among waste materials from the agri-food industry. They usually include fragments of plant biomass unsuitable for consumption left after the processing of cereals, vegetables, or fruits. Literature works present results of research into the sorption properties of such biomaterials as, e.g., fruit skins (bananas^1^, citrus fruits: oranges, mandarins, grapefruits, or lemons^2^, passion fruits^3^, apples^4^), or vegetable peels (of e.g. cucumbers, garlic^5^, potatoes^6^ or fava beans^7^). Other sorbents used for wastewater decolorization include leaves and stems of plants (mango^8^, banana tree^9^, maize^10^), nutshells (e.g. peanuts^11^, hazelnuts^12^, coconuts,^13^, walnuts, and almonds^14^), or husks of cereal grains (rice^15^, wheat^16^, or oats^17^). Most of the tested plant biomass-based sorbents showed very good sorption capability towards cationic dyes due to the high contents of polysaccharides and lignins. The greatest advantage of the waste plant biomass is, however, its common availability, which makes its inexpensive.

Examples of the widely available waste plant biomass include also spent coffee grounds and spent green tea leaves. Their vast availability stems from the extremely high popularity of beverages made of them (coffee and green tea), which are consumed in very large quantities. The annual global production of coffee (*Coffea arabica*) reaches 9–10 million tonnes^18^, whereas that of green tea approximates 2 million tonnes^19^. The relatively high contents of polysaccharides and lignins in the spent coffee grounds and spent green tea leaves (> 50% d.m.) indicate their potentially high capability for binding cationic dyes.

This study aimed to examine the sorption effectiveness of cationic dyes popular in the industry (Basic Red 46 and Basic Violet 10) onto spent coffee grounds and spent green tea leaves.

## Materials

### Raw materials used to produce sorbents

The sorbent based on the spent coffee grounds (CG) was produced from coffee (*Coffea arabica*) beans purchased at a local market. The coffee grains were “medium roasted” (10 min at a temp. of 230 °C) and then comminuted in a ceramic mortar to the particle size of 1–3 mm. The comminuted grains were brewed for 5 min (temp. of 90 °C). The average contents of cellulose, hemicellulose, and lignins in the brewed coffee (*Coffea arabica*) grains were: 12.4%, 39.1%, and 23.9%, respectively^20^.

The sorbent based on the spent green tea leaves (GTL) was prepared from leaves of Chinese green tea Bi Luo Chun (*Camellia sinensis var. sinensis*), purchased in a local tea shop. The leaves were brewed for 3 min at a temperature of 80 °C. The average contents of polysaccharides and lignins in the brewed green tea leaves were: cellulose—58.8%, hemicellulose—22.2%, and lignin—5.5%^21^.

Use of plants parts in the present study complies with international, national and/or institutional guidelines.

### Cationic dyes

The dyes were provided by a dye producing plant Boruta-Zachem SA (Zgierz, Poland). Their characteristics is presented in Table 1.

**Table 1:** Characteristics of Basic Red 46 and Basic Violet 10.

Name	Basic Red 46 (BR46)	Basic Violet 10 (BV10)
Structural formula		
Molecular weight	321.4 g/mol	479.0 g/mol
Dye class	azo dye (“single azo dye”)	xanthene dye
Absorption maximum (λ_max_)	530 nm (The calibration curve was prepared at pH 7)	554 nm (The calibration curve was prepared at pH 7)
Uses	Dyeing leather, paper, fabrics	Dyeing textiles, paper, leather
Harmfulness	Toxic, mutagenic, carcinogenic^22^	Toxic, fluorescent, carcinogenic; can induce skin and eye allergies, and gastrointestinal problems^23^

### Chemical reagents

The following chemical reagents were used in the study:

- Sulfuric acid 2 M solved solution (POCH S.A., Poland) − (sorbent preparation/purification).
- Hydrochloric acid 35—37% a.p. (POCH S.A., Poland) − (solution pH correction).
- Sodium hydroxide a.p. (POCH S.A., Poland) − (solution pH correction, sorbent preparation/purification).

### Laboratory equipment

The following laboratory equipment was used in the study:

- HI 110 pH meter, HANNA Instruments, Poland—(solution pH correction).
- Multi-Channel Stirrer MS-53M, JEIO TECH, Korea—(dye sorption analyses).
- UV-3100 PC spectrophotometer, VWR spectrophotometers, Canada—(determination of dye concentration in the solution).
- FT/IR-4700LE FT-IR Spectrometer with single reflection ATR attachment—JASCO INTERNATIONAL, Japan—(preparation of sorbent FTIR spectra).

## Methods

### Preparation of sorbents

The used, comminuted coffee grounds and green tea leaves were rinsed with deionized water (till colorless filtrate). Afterward, they were dried in a dryer (105 °C) and sieved through laboratory screens with mesh diameters of 1.0 and 2.0 mm. The fraction of grounds and leaves with particle diameters of 1–2 mm was kept in a 2 M solution of sulfuric VI acid for 24 h, then drained and washed with deionized water till the neutral pH of the filtrate. Afterward, the coffee grounds/green tea leaves were placed in a 2 M solution of sodium hydroxide for 24 h. Afterward, they were drained and rinsed with distilled water until neutral pH of the filtrate. Thus prepared spent coffee grounds (CG) and spent green tea leaves (GTL) were stored in a cooler at a temperature of 4 °C.

### Analyses of pH effect on dye sorption effectiveness

Sorbent doses of 1.00 g d.m. were weighed into a series of conical flasks (300 mL) using a technical scale. Then, earlier prepared dye solutions (200 mL) with the concentration of 50 mg/L and pH values of the subsequent solutions reaching: 2.0/3.0/4.0/5.0/6.0/7.0/8.0/9.0/10.0/11.0, were added to the flasks. The flasks with the solutions were placed on a magnetic stirrer (150 r.p.m.) for 120 min. Afterward, samples of the solutions (10 mL) were collected with an automatic pipette to earlier prepared test tubes. The dye concentration in the samples was determined with the spectrophotometric method (at the wavelengths of 554 nm for BV10 and 530 nm for BR46). Determinations were performed using 10 mm quartz measuring cuvettes. The calibration curve for both dyes was prepared at the pH 7. The solutions of dyes showed no changes both in UV–Vis absorbance and the position of the λ_max_ point at different pH values (in the range of pH 2–11 for BV10 and pH 2–8 for BR46) (Supplements 1, 2).

### Analysis of dye sorption kinetics

The sorbents (CG or GTL) were weighed in doses of 10.0 g d.m. into a series of beakers (2500 mL). Then, dye solutions (2000 mL) with sorption pH optimal for each dye (established based on analyses from “Analyses of pH effect on dye sorption effectiveness” section) and concentrations of: 50 mg/L, 200 mg/L, and 500 mg/L for Basic Red 46; or 10 mg/L, 50 mg/L, and 200 mg/L for Basic Violet 10, were added to the beakers. The beakers were placed on multi-station magnetic stirrers (150 r.p.m.). In the set time intervals (i.e., after: 0, 10, 20, 30, 45, 60, 90, 120, 150, 180, 210, 240, and 300 min), 5-mL samples were collected from the solutions with an automatic pipette. The dye concentration in the samples was determined with the method described in “Analyses of pH effect on dye sorption effectiveness” section.

### Analysis of the maximal sorption capacity

The sorbents (CG or GTL) were weighed in portions of 1.00 g d.m. to conical flasks (300 mL), which were also filled with dye solutions having the concentrations of 10–1000 mg/L (for BR46) or 10–500 mg/L (for BV10) and the optimal pH (established based on analyses performed in “Analyses of pH effect on dye sorption effectiveness” section). Next, the flasks were placed on a multi-station magnetic stirrer (150 r.p.m.) for the time needed to reach the sorption equilibrium (established based on the analyses performed in “Analysis of dye sorption kinetics” section). Once the sorption equilibrium had been reached, 10 mL of the solution was collected from the flasks using a pipette to determine the concentration of dye left in the solution after the sorption process.

### Calculation methods

The amount of dye bound on the sorbent was calculated from Eq. (1):

1 $$ {\text{Q}}_{{\text{S}}} = \left[ {\left( {{\text{C}}_{0} - {\text{C}}} \right) \times {\text{V}}} \right]/{\text{m}}. $$

Q_S_—mass of adsorbed dye [mg/g], C_0_ – initial dye concentration [mg/L], C—the concentration of dye left in the solution after the sorption process [mg/L], V—solution volume [L], M—sorbent mass [g].

The results of analysis on the kinetics of BR46 and BV10 sorption onto CG and GTL were described using the pseudo-first order model (2), pseudo-second order model (3), and the intramolecular diffusion model (4) (which allows determining the number and intensity of sorption phases).

2 $$ \Delta {\text{q}}/\Delta {\text{t}} = {\text{k}}_{{1}} \times \left( {{\text{q}}_{{\text{e}}} - {\text{q}}} \right), $$

3 $$ \Delta {\text{q}}/\Delta {\text{t}} = {\text{k}}_{{2}} \times \left( {{\text{q}}_{{\text{e}}} - {\text{q}}} \right)^{{2}} , $$

4 $$ {\text{q}} = {\text{k}}_{{{\text{id}}}} \times {\text{t}}^{0.5} . $$

q_e_—equilibrium amount of sorbed dye [mg/g], Q—instantaneous mass of adsorbed dye [mg/g], k_1_—sorption rate constant in the pseudo-first order model [1/min], k_2_—sorption rate constant in the pseudo-second order model [g/(min mg)], k_id_—sorption rate constant in the intramolecular diffusion model [mg/g min^0.5^], t—sorption time [min].

Experimental data from analysis of the maximal sorption capacity was described using: Langmuir 1 model (5), Langmuir 2 model (6), and Freundlich model (7).

5 $$ {\text{q}}_{{\text{e}}} = \left( {{\text{q}}_{{{\text{max}}}} \times {\text{K}}_{{\text{C}}} \times {\text{C}}} \right)/\left( {{1} + {\text{ K}}_{{\text{C}}} \times {\text{C}}} \right), $$

6 $$ {\text{q}}_{{\text{e}}} = \left( {{\text{b}}_{{1}} \times {\text{K}}_{{1}} \times {\text{C}}} \right)/\left( {{1} + {\text{ K}}_{{1}} \times {\text{C}}} \right) + \left( {{\text{b}}_{{2}} \times {\text{K}}_{{2}} \times {\text{C}}} \right)/\left( {{1} + {\text{K}}_{{2}} \times {\text{C}}} \right), $$

7 $$ {\text{q}}_{{\text{e}}} = {\text{K}} \times {\text{C}}^{{\text{n}}} . $$

q_e_—equilibrium amount of sorbed dye [mg/g], q_max_—maximal capacity of the monolayer [mg/g], b_1_—maximal capacity of type I active sites in the monolayer [mg/g], b_2_—maximal capacity of type II active sites in the monolayer [mg/g], K_1_/K_2_—constants in the double Langmuir equation [L/mg], K—sorption equilibrium constant in the Freundlich equation, C—the concentration of dye left in the solution after the sorption process [mg/L], n—heterogenicity parameter (Freundlich model).

## Results and discussion

### FTIR analysis of sorbents

**Figure 1:**
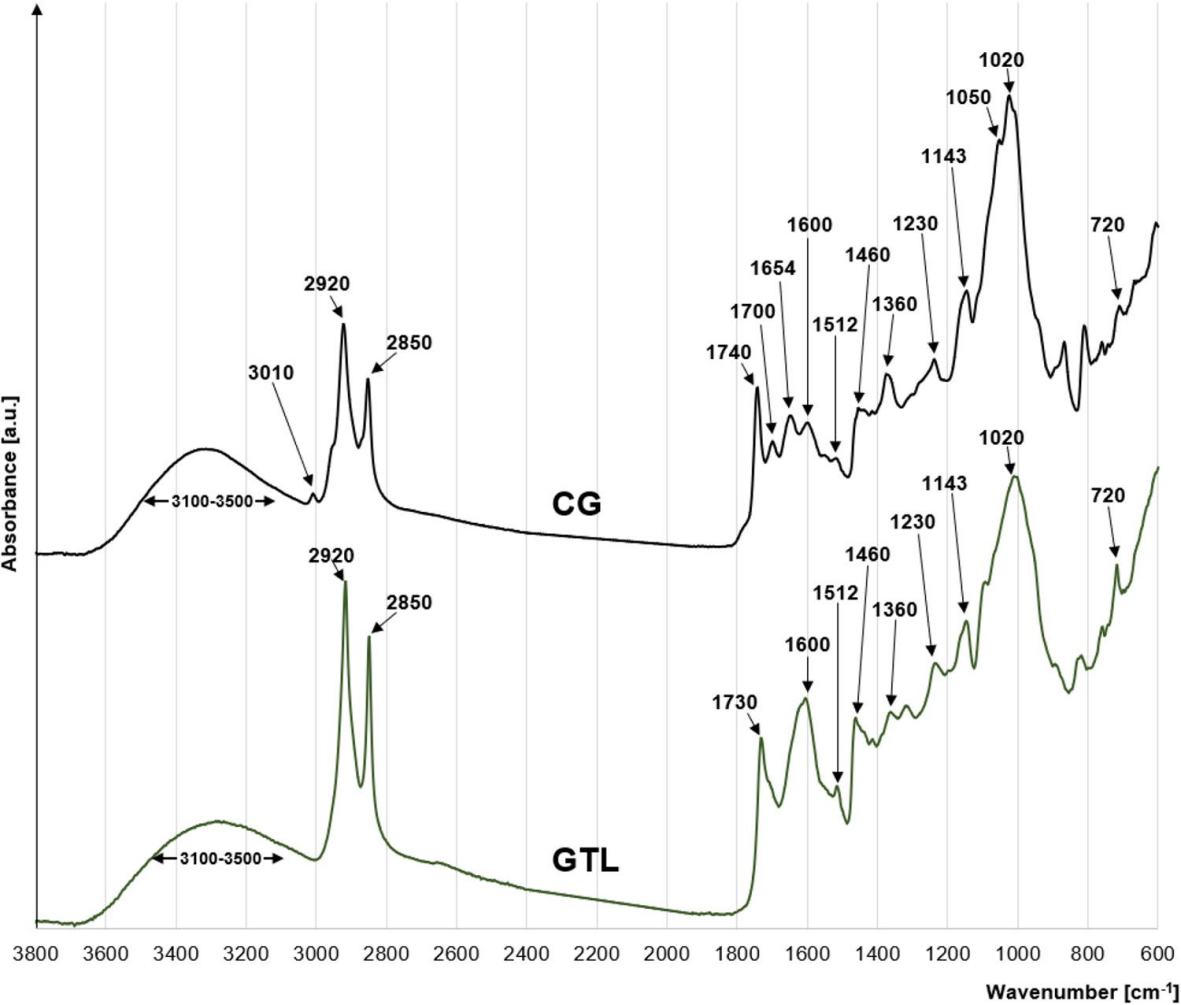
FTIR spectra of CG and GTL.

The CG and GTL spectra present peaks typical of the lignocellulosic plant biomass. A wide band at 3200–3400 cm^−1^ points to the stretching of the O–H bond of a hydroxyl group and to the presence of hydrogen bridges (Fig. 1). Distinct peaks at 2920 cm^−1^ and 2850 cm^−1^ correspond to the symmetrical stretching of –CH_3_ and asymmetrical stretching of –CH_2_–, and indicate lipid presence in material structure^24^. Peaks at 1730 cm^−1^ (GTL) and 1740 cm^−1^ (CG) correspond to the vibrations of a carbonyl bond (C=O) of the ester functional groups typical of hemicelluloses^25–27^. In turn, the peaks at 1600 cm^−1^, 1512 cm^−1^, and 1230 cm^−1^ are attributable to C=C bonds of guaiacol aromatic ring, typical of lignins^28^. The peaks noticeable at 1460 cm^−1^ (the bending of –CH_3_ and –CH_2_–), 1360 cm^−1^ (the asymmetrical bending of –CH_3_)^27,29^, and 720 cm^−1^ (the rocking/swinging of –CH_2_–) are typical of lignins and cellulose. In turn, the peaks at 1020 cm^−1^, 1050 cm^−1^, and 1143 cm^−1^ are attributable to C–O–C bond of the aromatic ring of polysaccharides^30^ (Fig. 1).

The peak visible at 3010 cm^−1^ only in the case of CG probably corresponds to the stretching of the =C–H bond, whereas the peak at 1700 cm^−1^ reflects the stretching of the C=O bond belonging to the carboxyl group of organic acid. The peak at 1654 cm^−1^ points to the presence of the N–H bond of caffeine^28^. In the case of GTL, the peak at 1654 cm^−1^ is, probably, obscured by a large peak at 1600 cm^−1^ (C=C bond) (Fig. 1).

### The effect of pH on the effectiveness of dye sorption onto CG and GTL

**Figure 2:**
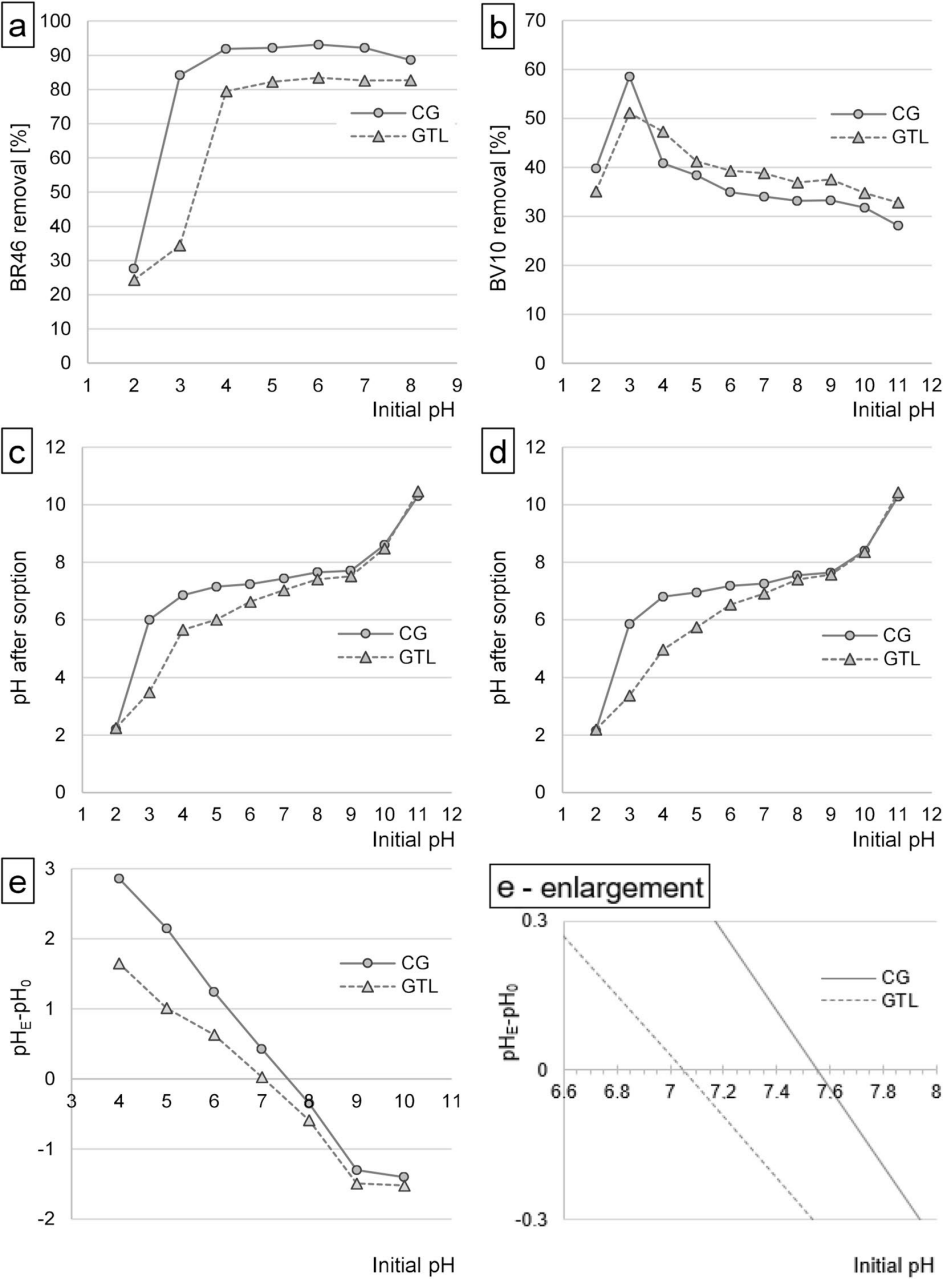
Effect of pH on the effectiveness of sorption of (**a**) BR46, and (**b**) BV10 onto CG and GTL. Effect of CG and GTL on changes in solution pH after sorption of: (**c**) BR46, and (**d**) BV10. (**e**)/e-enlargement)—Determination of pH_PZC_ of the sorbents (CG/GTL) with the Boehm’s titration method. Temp. 22 °C.

In the initial pH range of 2–6, the effectiveness of BR46 sorption onto CG and GTL increased along with pH increase, while at pH > 6 it decreased negligibly. The greatest changes in BR46 sorption effectiveness were noted at pH increase from pH 2 to pH 4, whereas at pH 4–8 the intensity of binding was similar (Fig. 2a). At pH > 8, BR46 decolorized spontaneously, which was verified in the preliminary study. For this reason, the results of BR46 sorption at pH 9–11 were not presented in Fig. 2a. The UV–Vis absorption spectra of the BR46 solution at pH 11 (5 min and 1440 min after preparing the solution) are shown in Supplement 3.

The relatively low effectiveness of BR46 sorption onto CG and GTL at low pH was due to the protonation of the functional groups of the tested sorbents (mainly the hydroxyl groups of polysaccharide and amine groups) and to the sorbent gaining a positive charge. The positively charged surface of sorbents repulsed electrostatically BR46 cations, which significantly impaired their sorption. Presumably, at pH > 4, the effectiveness of protonation of the functional groups of CG and GTL was low, which made the sorbent lose its positive charge. In addition, at pH > 4, a significant part of the acidic functional groups (e.g., carboxyl groups) underwent deprotonation, which made the sorbent gain a negative charge, thus boosting BR46 sorption effectiveness. A similar effect of pH on BR46 sorption effectiveness was also obtained in studies into dye sorption on coconut shells^13^, pumpkin seed husks^31^, and skins of citrus fruits^2^.

The BV10 sorption onto CG and GTL was the most effective at pH 3, and at the initial pH of 3–11 decreased along with pH increase. A significant decrease in BV10 sorption effectiveness was noted at pH < 3 (Fig. 2b). The untypical for cationic dyes effect of the pH value on the effectiveness of BV10 sorption onto CG and GTL was due to the acidic carboxyl functional group (–COOH) of the dye. In the pH range of 3–8, most of the carboxyl groups of the dye occurred in the deprotonated form (–COO^-^). Hence, despite generally alkaline character, BV10 molecules possessed a strong local negative charge. At low pH (pH 3), the positively-charged surface of the sorbent entered into the electrostatic interaction with deprotonated carboxyl groups of BV10, which enhanced dye sorption. The positive charge on sorbent surface decreased with pH increase, which weakened interactions with –COO– groups of the dye and ultimately contributed to a lower BV10 sorption effectiveness. Under alkaline conditions (pH > 8), the sorbent gained a negative charge, which resulted in the electrostatic repulsion between dye surface and –COO^-^ group of BV10, additionally inhibiting dye sorption. The lower BV10 sorption effectiveness onto CG and GTL at high pH could also be due to the competition between dye molecules and Na^+^ cations (pH correction using NaOH) for the sorption centers of biosorbents (mainly the hydroxyl groups of polysaccharides and lignins). The decrease in BV10 sorption effectiveness at pH < 3 could stem from the fact that at pH 2 nearly half of the carboxyl groups lose their negative charges in an aqueous solution (–COO^-^ + H_3_O^+^  → –COOH + H_2_O), which makes the electrostatic interactions with the sorbent far weaker. The diminished effectiveness of BV10 sorption onto CG and GTL at pH 2 could be significantly affected by the competition of –COO^-^ groups of the dye with Cl^-^ anions (pH correction using) for the active sites of the sorbent (mainly protonated hydroxyl groups).

The sorbent type (CG or GTL) did affect the dye solution pH during the sorption process (Fig. 2c,d). In the initial pH range of pH 4–9, after 2 h of dye sorption onto CG the pH value fixed at 6.9–7.6, whereas in the case of dye sorption onto GTL—at pH 5.7–7.6. The pH changes during sorption were due to the system tending to reach the pH value approximating the pH of the point of zero charge (pH_PZC_). The pH_PZC_ determined experimentally for CG and GTL reached pH_PZC_ = 7.55 and pH_PZC_ = 7.05 (Fig. 2e). The higher pH_PZC_ value determined for CG may stem from a higher number of alkaline functional groups (e.g., amine groups) of this sorbent.

### The kinetics of dye sorption onto CG and GTL

The time needed to reach BR46 and BV10 sorption equilibrium onto CG and GTL ranged from 180 to 240 min (Table 2, Supplement 4). A similar BR46 and BV10 sorption equilibrium time was noted in the study addressing dye sorption onto citrus fruit skins (180–240 min)^2^. In the case of BV10, similar sorption equilibrium times were also determined for such sorbents as: coconut shells (180 min)^13^, jute fiber (220 min)^32^, or activated carbon based on *Polygonum orientale* biomass (240 min)^33^.

**Table 2:** Kinetic parameters of BR46 and BV10 sorption onto CG and GTL determined from the pseudo-first order model and pseudo-second order model.

Sorbent	Dye	Dye conc	Equilibr. time	Exp. data	Pseudo-first order model			Pseudo-second order model		
				q_e. eksp_	k_1_	q_e_. _cal_	R^2^	k_2_	q_e_. _cal_	R^2^
		[mg/L]	[min]	[mg/g]	[1/min]	[mg/g]	–	[g/(mg min)]	[mg/g]	–
CG	BR46	50	240	9.36	0.0814	9.03	0.9933	0.0135	9.79	0.9981
		200	240	37.53	0.0684	35.58	0.9859	0.0026	39.12	0.9999
		500	240	88.36	0.0508	91.96	0.9784	0.0008	92.48	0.9986
	BV10	10	180	1.56	0.0505	1.39	0.9540	0.0443	1.57	0.9890
		50	240	7.05	0.0391	6.15	0.9600	0.0070	7.10	0.9889
		200	240	25.71	0.0373	21.91	0.9621	0.0018	25.44	0.9886
GTL	BR46	50	180	9.43	0.1225	9.97	0.9823	0.0240	9.52	0.9987
		200	240	30.80	0.0839	27.92	0.9568	0.0043	30.47	0.9912
		500	240	43.41	0.0710	39.51	0.9792	0.0024	43.51	0.9977
	BV10	10	240	1.72	0.0934	1.57	0.9681	0.0904	1.70	0.9954
		50	240	6.30	0.0696	5.52	0.9554	0.0172	6.09	0.9908
		200	240	15.01	0.0588	12.90	0.9480	0.0058	14.39	0.9867

The sorption of dyes onto CG and GTL was the most intensive in the first minutes of the process. As soon as in the first 30 min of the process, the amount of dyes bound onto CG ranged from 69.6 to 83.6% and from 58.5 to 66.5% of the q_e_ value (amount of dye sorbed after reaction equilibrium time) for BR46 and BV10, respectively. In the case of GTL, the amounts of BR46 and BV10 bound after 30 min were: 75.6–87.2% q_e_ and 64.6–78.5% q_e_.

The pseudo-second order model showed the best fit to the experimental data in each experimental series (Table 2). The q_e cal_ values (q_e_—equilibrium amount of sorbed dye) calculated from the models increased along with a higher initial dye concentration, which can be explained by the increasing likelihood of sorbate collisions with sorption centers. In contrast to q_e_, the values of sorption rate constants (k_2_) decrease along with dye concentration increase, typically of basic dye sorption onto biosorbents^2,31,34^.

**Figure 3:**
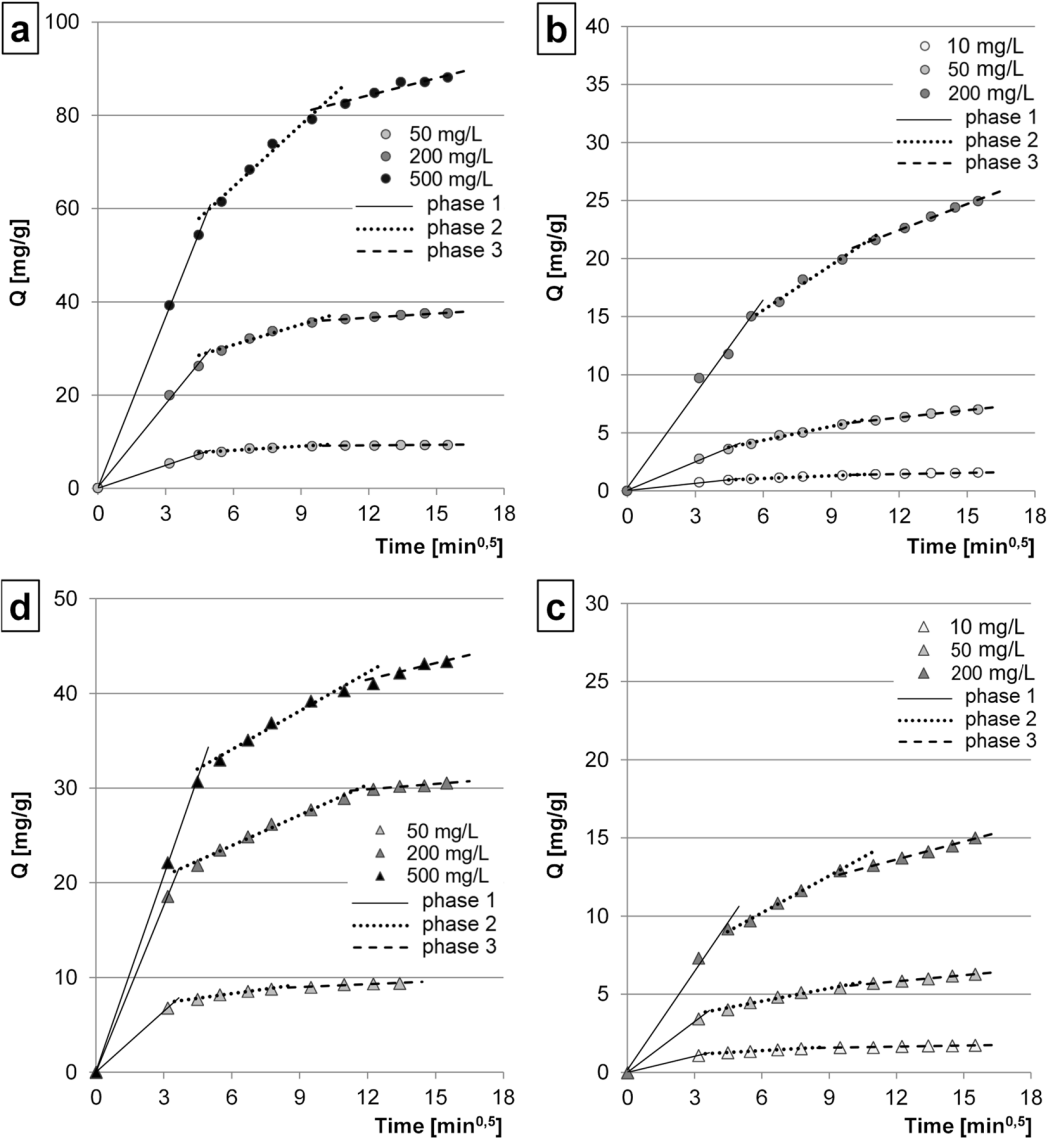
The intramolecular diffusion model of sorption of: (**a**) BR46 onto CG; (**b**) BV10 onto CG; (**c**) BR46 onto GTL; and (**d**) BV10 onto GTL. Temp. 22 °C.

In order to distinguish and describe particular phases of the sorption process, the experimental data obtained were described using the intramolecular diffusion model. The graphs plotted using this model suggest that the sorption of both BV10 and BR46 onto CG and GTL always proceeds in three phases (Fig. 3). In the first, the shortest, and the most intensive phase of the sorption, dye molecules probably attached to the most available active sites of the biosorbent, located mainly on its surface^34^. The second phase began once the easily available sorption centers depleted on the sorbent surface. In this phase, dye molecules occupied mainly the active sites located in the less accessible regions of the sorbent. In addition, this phase was much longer and characterized by a lower sorption intensity and by significantly greater competition between dye molecules for the active sites^35^, compared to the first phase. In the third, the longest and the least effective phase, the last free sorption centers were depleted (Fig. 3, Table 3).

**Table 3:** Rate constants of BR46 and BV10 diffusion determined from a simplified intramolecular diffusion model. Units: k_d1_, k_d2_, k_d3_ = [mg g^−1^ min^-0,5^], duration [min], R^2^ [–].

Sorbent	Dye	Dye conc. [mg/L]	Phase 1			Phase 2			Phase 3		
			k_d1_	Duration	R^2^	k_d2_	Duration	R^2^	k_d2_	Duration	R^2^
CG	BR46	50	1.636	~ 20	0.9989	0.298	~ 100	0.8942	0.040	~ 120	0.9431
		200	5.953	~ 20	0.9966	1.458	~ 100	0.9708	0.300	~ 120	0.9577
		500	12.196	~ 20	0.9997	4.442	~ 100	0.9767	1.230	~ 120	0.9191
	BV10	10	0.206	~ 20	0.9921	0.077	~ 70	0.9856	0.031	~ 150	0.9598
		50	0.816	~ 20	0.9950	0.397	~ 70	0.9696	0.214	~ 150	0.9761
		200	2.702	~ 30	0.9907	1.294	~ 60	0.9701	0.750	~ 150	0.9961
GTL	BR46	50	2.142	~ 10	0.9999	0.323	~ 50	0.9602	0.101	~ 120	0.8930
		200	5.867	~ 10	0.9999	1.077	~ 110	0.9856	0.188	~ 120	0.9530
		500	6.884	~ 20	0.9997	1.356	~ 100	0.9781	0.584	~ 120	0.8926
	BV10	10	0.346	~ 10	0.9999	0.079	~ 50	0.9831	0.024	~ 180	0.9770
		50	1.084	~ 10	0.9999	0.275	~ 80	0.9683	0.131	~ 150	0.9928
		200	2.103	~ 20	0.9909	0.790	~ 70	0.9961	0.381	~ 150	0.9918

The first key phase of dye sorption onto CG lasted 20 min in most experimental variants (Table 3), being longer (30 min) only at the highest BV10 concentration. In the case of GTL, the first phase of the sorption lasted from 10 min at the lower initial dye concentrations to 20 min at the higher initial concentrations of BR46/BV10. The increase in the k_d1_, k_d2_ and k_d3_ values observed in each sorption phase along with a higher initial concentration of both BR46 and BV10 was, presumably, due to the increasing availability of sorbate molecules in the solution and the higher likelihood of their collisions with the sorption centers.

The more effective sorption of BR46 onto CG and GTL compared to BV10 could stem from various sorption pHs and molecular weight of the dyes (BV10—479 g/mol, BR46—322 g/mol). The smaller sizes of Basic Red 46 molecules were probably reflected in its better capability for penetrating sorbent structure and reaching sorption centers located in its deeper, less available layers. The differences in dye sorption effectiveness could also be significantly affected by differences in their structure and various functional groups they possessed. Apart from methyl groups (–CH_3_) typical of BR46, BV10 possesses an acidic carboxyl group (–COOH^-^) untypical of cationic dyes, which makes it attain some features of anionic dyes.

### The maximal sorption capacity of CG and GTL

The experimental data, obtained in the analyses of the maximal sorption capacity, were described using popular sorption models, i.e.: Langmuir isotherm, double Langmuir isotherm (Langmuir 2 model), and Freundlich isotherm (Table 4, Supplement 5). The analysis of the coefficient of determination (R^2^) values indicated that the sorption of BR46 and BV10 onto CG and GTL was better described by the Langmuir and Langmuir 2 models than by the Freundlich model. The better fit of experimental data to the Langmuir and Langmuir 2 models than to the Freundlich model points to dye binding onto the sorbent in the form of a monolayer, where dye molecules may change their positions (i.e., “switch” their active sites).

**Table 4:** Constants determined from the Langmuir 1 model, Langmuir 2 model, and Freundlich model.

Sorbent	Dye	Langmuir 1			Langmuir 2						Freundlich		
		Q_max_	K_c_	R^2^	Q_max_	b_1_	K_1_	b_2_	K_2_	R^2^	K	n	R^2^
		[mg/g]	[L/mg]	–	[mg/g]	[mg/g]	[L/mg]	[mg/g]	[L/mg]	–	–	–	–
CG	BR46	165.85	0.022	0.995	179.36	99.47	0.036	79.89	0.008	0.996	14.2	0.43	0.950
	BV10	59.32	0.009	0.998	59.32	34.44	0.009	24.89	0.009	0.998	2.2	0.54	0.975
GTL	BR46	48.73	0.046	0.976	57.98	30.69	0.133	27.28	0.004	0.999	10.0	0.26	0.981
	BV10	23.41	0.016	0.978	26.66	22.73	0.017	3.93	0.004	0.998	2.2	0.38	0.987

The sorption of BR46 onto CG and GTL and the sorption of BV10 onto GTL were best described by the Langmuir 2 model, which implies an important role of more than one type of the active sites in the process or various modes of dye binding with the sorption center. Presumably, the hydroxyl functional groups derived from polysaccharides (cellulose/hemicellulose), located on the sorbent surface, served as the first type of the active sites. In turn, both amine groups and hydroxyl groups located in deeper, less accessible sorbent layers, could serve as the second type of sorption centers. A significant role in BR46 sorption could also be ascribed to the carboxyl groups. In addition, the dyes tested could bind with the functional groups of the sorbents also by means of hydrogen bridges. In case of BV10 sorption onto CG, the K_c_ constants computed from the Langmuir model and the K_1_ and K_2_ constants calculated from the Langmuir 2 model have the same values, which may suggest that one type of active sites was responsible for this dye sorption onto CG. Presumably, this role was played by the protonated amine groups of CG. No similar result was observed for GTL due to a lower number of alkaline (amine) functional groups, as mentioned during the analysis of the pH_PZC_ values of the sorbents (“The effect of pH on the effectiveness of dye sorption onto CG and GTL” section).

Compared to BV10, BR46 showed generally greater affinity to the functional groups of CG and GTL, as evidenced by higher values of K_c_, K_1_, and K_2_ constants. The sorbents tested also show higher sorption capacity of BR46 than of BV10 (Table 4). As mentioned in “The kinetics of dye sorption onto CG and GTL” section, the differences observed in dye sorption capacity could be due the various nature of functional groups of the dyes, different sorption conditions (pH 3 for BV10 and pH 6 for BR46), and also various molecular weights of dyes (BV10—479 g/mol, BR46—322 g/mol). The smaller dimensions of BR46 molecules facilitate sorbent structure penetration and occupation of a higher number of sorption centers available in the sorbent, which explains higher BR46 sorption effectiveness.

The better BR46 sorption properties of CG compared to GTL could be attributed to a higher number of carboxyl functional groups, as demonstrated during FTIR spectra analysis (“The kinetics of dye sorption onto CG and GTL” section). In turn, the higher effectiveness of BV10 sorption onto CG than GTL could be due to a higher number of alkaline (amine) functional groups on CG, which was confirmed by the pH_PZC_ values of the sorbents (“The effect of pH on the effectiveness of dye sorption onto CG and GTL” section). The higher sorption capacity of CG than of GTL can also stem from the partial carbonization of coffee grain during their “roasting”, which could contribute to significant enlargement of sorbent surface, thereby increasing its sorption capacity.

Table 5 collates sorption capacities of various unconventional sorbents and activated carbons towards dyes tested in this research, i.e. BR46 and BV10. The sorbents analyzed in the present study (CG and GTL) demonstrated higher sorption capacity of cationic dyes BR46 and BV10 than biosorbents based on fruit skins, tree leaves, and sawdust (Table 5). What is more, dye sorption effectiveness was higher on CG compared to activated carbons produced from lignocellulosic plant biomass^32,36,37^ and was inferior only to the high-quality commercial activated carbon^38^.

**Table 5:** Sorption capacity of various unconventional biosorbents and activated carbons towards BV10 and BR46.

Dye	Sorbent	Sorption capacity [mg/g]	Sorption pH [pH]	Sorption time [min]	Source
BR46	Beech sawdust	19.2	–	–	^39^
	Fir sawdust	20.5	–	–	^39^
	Walnut sawdust	30.1	7	–	^40^
	*Paulownia tomentosa* tree leaves	43.1	8	70	^22^
	Active carbon ROW 08	45.0	8	60	^41^
	Rape seed husks	49.0	8	10	^42^
	Lemon skin	54.0	6	240	^2^
	Mandarin skin	54.2	6	240	^2^
	Spent green tea leaves	58.0	6	240	This work
	Active carbon from the *Cerbera* biomass	65.7	7	90	^36^
	Coconut shells	68.5	6	120	^13^
	Pine leaves	71.9	6	75	^43^
	Pine cones	73.5	8	75	^44^
	Spent coffee grounds	179.4	6	240	This work
BV10	Coal-fired coconut fiber	2.6	6.5	150	^45^
	Mango leaves (powder)	3.3	–	48	^46^
	*Calotropis procera* leaf biomass	4.1	–	60	^47^
	Cedar cones	4.6	–	360	^39^
	Grapefruit skin	4.6	3	240	^2^
	Lemon skin	5.7	3	240	^2^
	Sugar cane fiber	10.4	–	–	^48^
	Coconut fiber	16.5	7	90	^49^
	Active fiber from walnut shell	18.7	–	–	^37^
	Banana skin	20.6	6	1440	^50^
	Spent green tea leaves	26.7	3	240	This work
	Active carbon from jute fiber	28.0	8	220	^32^
	Coconut shells	28.5	3	180	^13^
	Active carbon from palm bark	30.0	3	–	^51^
	Spent coffee grounds	59.3	3	240	This work
	Commercial active carbon powder	72.5	4	1440	^38^

## Summary

Appropriately prepared spent coffee ground and spent green tea leaves may serve as effective sorbents of cationic dyes. In the case of dyes tested, i.e. BR46 and BV10, the maximal sorption capacity of these sorbents reached: 179.4 mg/g and 59.3 mg/g for CG and 58.0 mg/g and 26.7 mg/g for GTL, respectively.

The effectiveness of BV10 and BR46 binding onto CG and GTL was significantly determined by the pH value of the sorption solution. Despite the similar nature of both dyes (basic dyes), they required various pH values to reach the maximum sorption capacity. The sorption of BV10 onto CG and GTL was the most effective at pH 3, whereas that of BR46—at pH 6. The relatively low pH value of BV10 dye sorption (pH 3) was due to its acidic carboxyl functional group (–COOH). Both CG and GTL caused changes in solution pH during the sorption process, due to the system tending to reach the pH value approximating the pH zero point charge (pH_PZC_ = 7.55 for CG and pH_PZC_ = 7.05 for GTL).

The effectiveness of BR46 sorption onto CG and GTL was higher than that of BV10, which was due to the various functional groups of the dyes, different sorption pHs, and different molecular weights of the dyes (BR46—322 g/mol, BV10—479 g/mol). The smaller molecules of BR46 compared to BV10 allowed its easier penetration into sorbent structure and its access to the less available sorption centers.

The better sorption capabilities of GC compared to GTL can be due to a higher number of carboxyl functional groups and greater specific area gained during coffee grain “roasting”.

The time needed to reach BR46 and BV10 sorption equilibrium onto CG and GTL was similar and ranged from 180 to 240 min. However, the intensity of dye sorption increased along with a higher initial dye concentration, especially in the first minutes of the process. This could be attributed to a high number of dye molecules in the solution and higher probability of their collisions with sorption centers.

The experimental data concerning the kinetics of BR46 and BV10 sorption onto CG and GTL were the best described with the pseudo-second order model, which is typical of the sorption of cationic dyes on biosorbents.

The sorption of BR46 and BV10 onto CG and GTL proceeded in three main phases. In the first, the shortest, and the most intensive phase, dye molecules occupied the most available sorption sites located mainly on sorbent surface. In the second, much longer, and less intensive phase, the saturation of the sorption centers was mainly observed in less available sorbent regions. In the third, the longest, and the least intensive phase, dye molecules slowly attached to the last free active sites of the sorbent.

The sorption of BR46 and BV10 onto CG and GTL was better described by the Langmuir 1 and Langmuir 2 models than by the Freundlich model, which points to dye binding onto the sorbent in the form of a monolayer, where dye molecules may change their positions (they can “switch” their active sites). The better fit of the Langmuir 2 than the Langmuir 1 model to the experiment data of BR46 sorption onto CG and GTL and BR46 sorption onto GTL implies an important role of more than one type of the active sites in the process or various modes of dye binding with the sorption center. Hydroxyl, carboxyl, and amine group are the active sites of sorbents that may play a significant role in the sorption process of cationic dyes. In the case of BV10 sorption onto CG, the K_c_ constants computed from the Langmuir model and the K_1_ and K_2_ constants computed from the Langmuir 2 model had the same values, which may suggest that only one type of active sites (probably the protonated amine groups) was responsible for its sorption onto CG.

## Supplementary Information

Supplementary Information.

## References

[1] Annadurai G, Juang RS, Lee DJ (2002). Use of cellulose-based wastes for adsorption of dyes from aqueous solutions. J. Hazard. Mater..

[2] Jóźwiak T, Filipkowska U, Zajko P (2019). Use of citrus fruit peels (grapefruit, mandarin, orange, and lemon) as sorbents for the removal of Basic Violet 10 and Basic Red 46 from aqueous solutions. Desalin. Water Treat..

[3] Nhung NTH, Quynh BTP, Thao PTT, Bich HN, Giang BL (2018). Pretreated fruit peels as adsorbents for removal of dyes from water. IOP Conf. Ser. Earth Environ. Sci..

[4] Enniya I, Jourani A (2017). Study of methylene blue removal by a biosorbent prepared with apple peels. J. Mater. Environ. Sci..

[5] Stavrinou A, Aggelopoulos CA, Tsakiroglou CD (2018). Exploring the adsorption mechanisms of cationic and anionic dyes onto agricultural waste peels of banana, cucumber and potato: Adsorption kinetics and equilibrium isotherms as a tool. J. Environ. Chem. Eng..

[6] Bouhadjra K, Lemlikchi W, Ferhati A, Mignard S (2021). Enhancing removal efficiency of anionic dye (Cibacron blue) using waste potato peels powder. Sci. Rep..

[7] Bayomie OS (2020). Novel approach for effective removal of methylene blue dye from water using fava bean peel waste. Sci. Rep..

[8] Vyavahare G (2019). Strategies for crystal violet dye sorption on biochar derived from mango leaves and evaluation of residual dye toxicity. J. Clean. Prod..

[9] Hameed BH, Mahmoud DK, Ahmad AL (2008). Sorption equilibrium and kinetics of basic dye from aqueous solution using banana stalk waste. J. Hazard. Mater..

[10] Crini G (2006). Non-conventional low-cost adsorbents for dye removal: A review. Bioresour. Technol..

[11] Etorki AM, Massoudi FMN (2011). The use of peanut hull for the adsorption of colour from aqueous dye solutions and dye textile effluent. Orient. J. Chem..

[12] Kaya N, Yıldız Z, Ceylan S (2018). Preparation and characterisation of biochar from hazelnut shell and its adsorption properties for methylene blue dye. J. Polytech..

[13] Józwiak T, Filipkowska U, Bugajska P, Kalkowski T (2018). The use of coconut shells for the removal of dyes from aqueous solutions. J. Ecol. Eng..

[14] Kocaman S (2020). Removal of methylene blue dye from aqueous solutions by adsorption on levulinic acid-modified natural shells. Int. J. Phytoremediat..

[15] Quansah JO (2020). Nascent rice husk as an adsorbent for removing cationic dyes from textile wastewater. Appl. Sci..

[16] Das S, Singh S, Garg S (2019). Evaluation of wheat bran as a biosorbent for potential mitigation of dye pollution in industrial waste waters. Orient. J. Chem..

[17] Banerjee S (2016). Removal of Malachite Green, a hazardous dye from aqueous solutions using *Avena sativa* (oat) hull as a potential adsorbent. J. Mol. Liq..

[18] Gruczyńska E, Kowalska D, Kozłowska M, Majewska E, Tarnowska K (2018). Furan in roasted, ground and brewed coffee. Rocz. Panstw. Zakl. Hig..

[19] Prasanth MI, Sivamaruthi BS, Chaiyasut C, Tencomnao T (2019). A review of the role of green tea (*Camellia sinensis*) in antiphotoaging, stress resistance, neuroprotection, and autophagy. Nutrients.

[20] Ballesteros LF, Teixeira JA, Mussatto SI (2014). Chemical, functional, and structural properties of spent coffee grounds and coffee silverskin. Food Bioprocess. Technol..

[21] Rahman NHA, Chieng BW, Ibrahim NA, Rahman NA (2017). Extraction and characterization of cellulose nanocrystals from tea leaf waste fibers. Polymers (Basel).

[22] Deniz F, Saygideger SD (2011). Removal of a hazardous azo dye (Basic Red 46) from aqueous solution by princess tree leaf. Desalination.

[23] Hassani A (2018). Enhanced removal of basic violet 10 by heterogeneous sono-Fenton process using magnetite nanoparticles. Ultrason. Sonochem..

[24] Sivakumar S (2014). FT-IR study of green tea leaves and their diseases of Arunachal Pradesh, North East, India. Pharm. Chem. J..

[25] Li X (2018). Rapid determination of chlorophyll and pheophytin in green tea using fourier transform infrared spectroscopy. Molecules.

[26] Craig AP, Franca AS, Oliveira LS (2012). Evaluation of the potential of FTIR and chemometrics for separation between defective and non-defective coffees. Food Chem..

[27] Lionetto F, Del Sole R, Cannoletta D, Vasapollo G, Maffezzoli A (2012). Monitoring wood degradation during weathering by cellulose crystallinity. Materials (Basel)..

[28] Monje AFB, Parrado LX, Gutiérrez-Guzmán N (2018). ATR-FTIR for disc rimenation of espresso and americano coffee pods. Coffee Sci..

[29] Zhang ZP, Rong MZ, Zhang MQ, Yuan C (2013). Alkoxyamine with reduced homolysis temperature and its application in repeated autonomous self-healing of stiff polymers. Polym. Chem..

[30] Nandiyanto ABD, Oktiani R, Ragadhita R (2019). How to read and interpret ftir spectroscope of organic material. Indones. J. Sci. Technol..

[31] Kowalkowska A, Jóźwiak T (2019). Utilization of pumpkin (*Cucurbita pepo*) seed husks as a low-cost sorbent for removing anionic and cationic dyes from aqueous solutions. Desalin. Water Treat..

[32] Porkodi K, Vasanth Kumar K (2007). Equilibrium, kinetics and mechanism modeling and simulation of basic and acid dyes sorption onto jute fiber carbon: Eosin yellow, malachite green and crystal violet single component systems. J. Hazard. Mater..

[33] Wang L (2010). Adsorption of basic dyes on activated carbon prepared from *Polygonum orientale* Linn: Equilibrium, kinetic and thermodynamic studies. Desalination.

[34] Kumar R, Ahmad R (2011). Biosorption of hazardous crystal violet dye from aqueous solution onto treated ginger waste (TGW). Desalination.

[35] Kulkarni MR, Revanth T, Acharya A, Bhat P (2017). Removal of crystal violet dye from aqueous solution using water hyacinth: Equilibrium, kinetics and thermodynamics study. Resour. Technol..

[36] Azmi NAI, Zainudin NF, Ali UFM (2015). Adsorption of basic Red 46 using sea mango (*Cerbera odollam*) based activated carbon. AIP Conf. Proc..

[37] Sumanjit Walia TPS, Kansal I (2008). Removal of Rhodamine-B by adsorption on walnut shell charcoal. J. Surf. Sci. Technol..

[38] Filipkowska U, Jόźwiak T, Szymczyk P, Kuczajowska-Zadrożna M (2017). The use of active carbon immobilised on chitosan beads for RB5 and BV10 dye removal from aqueous solutions. Prog. Chem. Appl. Chitin its Deriv..

[39] Zamouche M, Hamdaoui O (2012). Sorption of Rhodamine B by cedar cone: Effect of pH and ionic strength. Energy Procedia.

[40] Yeddou N, Bensmaili A (2005). Kinetic models for the sorption of dye from aqueous solution by clay-wood sawdust mixture. Desalination.

[41] Madeła MD, Krzemińska EN (2014). Wpływ procesu Fentona na skuteczność usuwania zanieczyszczeń ze ścieków przemysłowych na węglach aktywnych. Technol. Wody.

[42] Mahmoodi NM, Arami M, Bahrami H, Khorramfar S (2010). Novel biosorbent (Canola hull): Surface characterization and dye removal ability at different cationic dye concentrations. Desalination.

[43] Deniz F, Karaman S, Saygideger SD (2011). Biosorption of a model basic dye onto *Pinus brutia* Ten.: Evaluating of equilibrium, kinetic and thermodynamic data. Desalination.

[44] Deniz F, Karaman S (2011). Removal of basic red 46 dye from aqueous solution by pine tree leaves. Chem. Eng. J..

[45] Namasivayam C (2001). ‘Waste’ coir pith—A potential biomass for the treatment of dyeing wastewaters. Biomass Bioenergy.

[46] Hussain-Gardazi SM (2016). Effective adsorption of cationic dye from aqueous solution using low-cost corncob in batch and column studies. Desalin. Water Treat..

[47] Ali H, Muhammad SK (2008). Biosorption of crystal violet from water on leaf biomass of *Calotropis procera*. J. Environ. Sci. Technol..

[48] Parab H, Sudersanan M, Shenoy N, Pathare T, Vaze B (2009). Use of agro-industrial wastes for removal of basic dyes from aqueous solutions. Clean: Soil, Air, Water.

[49] Sureshkumar MV, Namasivayam C (2008). Adsorption behavior of Direct Red 12B and Rhodamine B from water onto surfactant-modified coconut coir pith. Colloids Surf. A Physicochem. Eng. Asp..

[50] Namasivayam C, Kanchana N, Yamuna RT (1993). Waste banana pith as adsorbent for the removal of rhodamine-B from aqueous solutions. Waste Manage..

[51] Mohammadi M, Hassani AJ, Mohamed AR, Najafpour GD (2010). Removal of rhodamine b from aqueous solution using palm shell-based activated carbon: Adsorption and kinetic studies. J. Chem. Eng. Data.

