# Supplementary material for: The use of spent coffee grounds and spent green tea leaves for the removal of cationic dyes from aqueous solutions
Source: Sci Rep. 2021 May 5;11:9584. doi: 10.1038/s41598-021-89095-6 (PMC8100174; doi:10.1038/s41598-021-89095-6)
Supplement: Supplementary file 1 — Supplementary Information. [file 41598_2021_89095_MOESM1_ESM.docx]

**The use of spent coffee grounds and spent green tea leaves for the removal of cationic dyes from aqueous solutions**

Tomasz Jóźwiak^1a*^, Urszula Filipkowska^1b^, Joanna Struk-Sokołowska^2c^,
Kamil Bryszewski ^1d^, Karol Trzciński^1e^, Joanna Kuźma^1f^, Monika Ślimkowska^1g^

^1^ Department of Environmental Engineering, University of Warmia and Mazury in Olsztyn, Warszawska St. 117a, 10-957 Olsztyn, Poland

^2^ Department of Environmental Engineering Technology, Bialystok University of Technology, Wiejska St. 45E, Bialystok 15-351, Poland

^a^ tomasz.jozwiak@uwm.edu.pl, ^b^ urszula.filipkowska@uwm.edu.pl,  ^c^ [j.struk@pb.edu.pl](mailto:j.struk@pb.edu.pl),

^d^ kamil.bryszewski@uwm.edu.pl, ^e^ karol.trzcinski@gmail.com,
^f^ joanna.ulewicz96@gmail.com, ^g^ monika.slimkowska@gmail.com

* Corresponding author: Tomasz Jóźwiak, Department of Environmental Engineering, University of Warmia and Mazury in Olsztyn, ul. Warszawska 117a, 10-957 Olsztyn, Poland, e-mail: tomasz.jozwiak@uwm.edu.pl

**Supplementary Data**

Suppl. 1. The UV-VIS absorption spectra of the BV10 solution (initial conc. = 10.0 mg/L) at different pH: a) pH 2; b) pH 6; c) pH 11, before and after sorption on GTL (dose of sorbent = 1 g/L; sorption time = 120 min). Temp. 22 ^o^C.

Suppl. 2. The UV-VIS absorption spectra of the BR46 solution (initial conc. = 50.0 mg/L) at different pH: a) pH 2; b) pH 6; c) pH 11, before and after sorption on GTL (dose of sorbent = 1 g/L; sorption time = 120 min). Temp. 22 ^o^C.

Suppl. 3. The UV-VIS absorption spectra of the BR46 solution (initial conc. = 50.0 mg/L) at pH 11 (5 min and 1440 min after preparing the solution). Temp. 22 ^o^C.

Suppl. 4. Kinetics of sorption of: a) BR46 onto CG; b) BV10 onto CG; c) BR46 onto GTL; and d) BV10 onto GTL. The pseudo-first order model and the pseudo-second order model. Temp. 22 ^o^C.

Suppl. 5. Isotherms of sorption of: a) BR46 onto CG; b) BV10 onto CG; c) BR46 onto GTL; and d) BV10 onto GTL. Temp. 22 ^o^C.
